# A Ministry of Health

**Published:** 1917-03-31

**Authors:** 


### The Hospital

The Workers' Newspaper of Administrative Medicine and Institutional Life, Administration, National Insurance and Health.

No. 1608, Vol. LXI. SATURDAY, MARCH 31, 1917. PRICE ONE PENNY

### A MINISTRY OF HEALTH.

In these days of rapidly evolved new official Directors and authoritative Controllers it is hardly surprising to find a revival of the claim for the establishment of a Ministry of Health. The war, here as in so many other directions, adds force to the suggestion, as it has set in a bright light the relation between the vigour of the people and the national struggle for existence. Add to this consideration the sad fact that our young manhood is being sacrificed in thousands, and it is little wonder that prudent and thoughtful minds recognise the need for a survey of the methods by which the invasions of disease may be opposed and resisted. Again, it is an outworn cry that in matters of health each citizen may be his own custodian and arbiter. Individualism in this respect has yielded to the imperative claims of necessity, and the public conscience demands that both imperial and municipal legislation and administration shall concern themselves with measures directed to the conservation of the health of the community. Whether the ills to which flesh is heir are or are not punishments devised by the gods, the demand is universal that medical and sanitary science shall be organised to check and overcome them. What was at one time regarded as an act of impiety is now claimed as practical prudence and common sense. As the growth of this new view of things has been gradual, the measures in which it has found expression have equally been partial and limited. Here, as elsewhere, it has been impossible for legislation to move substantially in advance of public opinion. Hence many of the arrangements which as a matter of fact are to-day concerned with the care of the public health are the product of legislation dealing with individual emergencies. Even allowing that each one of them is well devised for its immediate purpose, it is obvious that in such circumstances there will be inevitable weaknesses. In some directions there will be overlapping; in others, gaps; and throughout co-ordination and co-operation will be conspicuous by their absence. When the Home Office, the Local Government Board, the Education Department, the Insurance Commission, and the Poor Law divide between them the care of the health of the people no one experienced in the ways of officials will anticipate either smooth working or effective results. This is the actual situation which presents itself to a public opinion forced by the pressure of events to appraise the importance of health in a national scheme of things. Obviously the situation is one favourable to the arguments of those who, without much encouragement, urged in more placid times the establishment of a Ministry of Health. The Hospital has taken its share in this advocacy, and welcomes the new activity which the movement displays. Whatever be the machinery selected, it is the plainest patriotism to claim that a more efficient care of the health of the community must be part of the scheme under which the country is governed.

Judging from the recent discussion at the interview granted by Lord Rhondda to a deputation appointed by the British Medical Association there is no early prospect of any official attempt to re-adjust the existing state of things. The Local Government Board is at the moment concerned with a scheme dealing with maternity and infant welfare, and as the President entertains the expectation that by this scheme 50,000 young lives may be saved every year he can hardly be asked to postpone his plans until a Ministry of Health has been devised and elaborated. For this latter task will certainly take time, and there are many lions in the path. Hence, very naturally, the Board is determined on what it is convinced is an immediate benefit, and with this determination we are by no means disposed to quarrel. When the official scheme is produced we shall be in a position to verify its virtues, and if these are equal to the advance claims everyone will wish it Godspeed. Its existence, however, will not in any direct fashion help towards the Ministry of Health, and indeed, considered from this point of view, it will add one more department to those which already exist, and which, as is generally admitted, need to be brought into some organic relation with one another if the problem of the public health is to be placed in the position which its importance demands.

What the medical deputation was specially concerned with was the relation of the general medical practitioner to the official proposals. Lord Rhondda expressed the view that the practitioner entertained some feeling of suspicion towards his Board, and he desired to overcome this. For our own part we think the general practitioner has some grounds for looking suspiciously, we will not say on the Local Government Board in particular, but certainly on some proposals which enjoy official sanction. A number of these invade his area of work and opportunity and responsibility, and they do so often with a complete disregard of his special experience and competence. If the results were a triumph for the public interest the practitioner must submit to be sacrificed as a factor of minor importance. But personally we doubt this triumph, and we entertain a strong view that if the full benefits of medical knowledge and capacity, whether preventive or curative, are to be brought into the homes and lives of our people the general medical practitioner will have to play an important part in the undertaking. It is because this principle has been ignored in the past that certain official schemes have proved ineffective, and the lesson is an important one for those who hope to co-ordinate all existing departments under the control of one supreme Ministry of Health.

